# Supplementary material for: FastqPuri: high-performance preprocessing of RNA-seq data
Source: BMC Bioinformatics. 2019 May 3;20:226. doi: 10.1186/s12859-019-2799-0 (PMC6500068; doi:10.1186/s12859-019-2799-0)
Supplement: Supplementary file 2 — Archive of FastqPuri. Archive containing all files needed to install and run FastqPuri v1.0.6. Date stamp March 22, 2019. (GZ 47,819 kb) [file 12859_2019_2799_MOESM2_ESM.gz › FastqPuri-1.0.6/html/stats__info_8h_source.html]

FastqPuri: include/stats\_info.h Source File


FastqPuri


- include

stats\_info.h

Go to the documentation of this file.

1 /\*\*\*\*\*\*\*\*\*\*\*\*\*\*\*\*\*\*\*\*\*\*\*\*\*\*\*\*\*\*\*\*\*\*\*\*\*\*\*\*\*\*\*\*\*\*\*\*\*\*\*\*\*\*\*\*\*\*\*\*\*\*\*\*\*\*\*\*\*\*\*\*\*\*\*\*

2  \* Copyright (C) 2017 by Paula Perez Rubio \*

3  \* \*

4  \* This file is part of FastqPuri. \*

5  \* \*

6  \* FastqPuri is free software: you can redistribute it and/or modify \*

7  \* it under the terms of the GNU General Public License as \*

8  \* published by the Free Software Foundation, either version 3 of the \*

9  \* License, or (at your option) any later version. \*

10  \* \*

11  \* FastqPuri is distributed in the hope that it will be useful, \*

12  \* but WITHOUT ANY WARRANTY; without even the implied warranty of \*

13  \* MERCHANTABILITY or FITNESS FOR A PARTICULAR PURPOSE. See the \*

14  \* GNU General Public License for more details. \*

15  \* \*

16  \* You should have received a copy of the GNU General Public License \*

17  \* along with FastqPuri. \*

18  \* If not, see <http://www.gnu.org/licenses/>. \*

19  \*\*\*\*\*\*\*\*\*\*\*\*\*\*\*\*\*\*\*\*\*\*\*\*\*\*\*\*\*\*\*\*\*\*\*\*\*\*\*\*\*\*\*\*\*\*\*\*\*\*\*\*\*\*\*\*\*\*\*\*\*\*\*\*\*\*\*\*\*\*\*\*\*\*\*\*/

20

29 #ifndef STATS\_INFO\_H\_

30 #define STATS\_INFO\_H\_

31

32 #include <stdint.h>

33 #include <stdlib.h>

34 #include "fq\_read.h"

35 #include "defines.h"

36

37

41 typedef struct statsinfo {

42  int read\_len;

43  int ntiles;

44  int nQ;

45  int minQ;

46  int tile\_pos;

47  int nreads;

48  int reads\_wN;

49  int sz\_lowQ\_ACGT\_tile;

50  int sz\_ACGT\_tile;

51  int sz\_reads\_MlowQ;

52  int sz\_QPosTile\_table;

53  int sz\_ACGT\_pos;

54  int \*tile\_tags;

55  int \*lane\_tags;

56  int \*qual\_tags;

57  uint64\_t\* lowQ\_ACGT\_tile;

58  uint64\_t\* ACGT\_tile;

61  uint64\_t\* reads\_MlowQ;

63  uint64\_t\* QPosTile\_table;

64  uint64\_t\* ACGT\_pos;

65 } Info;

66

67 void init\_info(Info\* res);

68 void free\_info(Info\* res);

69 void read\_info(Info\* res, char\* file);

70 void write\_info(Info\* res, char\* file);

71 void print\_info(Info\* res, char \*infofile);

72

73 void get\_first\_tile(Info\* res, Fq\_read\* seq);

74 void update\_info(Info\* res, Fq\_read\* seq);

75 int update\_ACGT\_counts(uint64\_t\* ACGT\_low, char ACGT);

76 void update\_QPosTile\_table(Info \*res, Fq\_read \*seq);

77 void update\_ACGT\_pos(uint64\_t\* ACGT\_pos, Fq\_read \*seq);

78 void resize\_info(Info\* res);

79

80 #endif // endif STATS\_INFO\_H\_

statsinfo::ACGT\_tile

uint64\_t \* ACGT\_tile

**Definition:** stats\_info.h:58

statsinfo::minQ

int minQ

**Definition:** stats\_info.h:45

statsinfo::sz\_ACGT\_tile

int sz\_ACGT\_tile

**Definition:** stats\_info.h:50

init\_info

void init\_info(Info \*res)

Initialization of a Info type.

**Definition:** stats\_info.c:103

get\_first\_tile

void get\_first\_tile(Info \*res, Fq\_read \*seq)

gets first tile

**Definition:** stats\_info.c:312

statsinfo::ntiles

int ntiles

**Definition:** stats\_info.h:43

statsinfo::sz\_QPosTile\_table

int sz\_QPosTile\_table

**Definition:** stats\_info.h:52

update\_info

void update\_info(Info \*res, Fq\_read \*seq)

updates Info with Fq\_read

**Definition:** stats\_info.c:319

statsinfo::reads\_wN

int reads\_wN

**Definition:** stats\_info.h:48

\_fq\_read

stores a fastq entry

**Definition:** fq\_read.h:37

statsinfo::sz\_lowQ\_ACGT\_tile

int sz\_lowQ\_ACGT\_tile

**Definition:** stats\_info.h:49

statsinfo::lane\_tags

int \* lane\_tags

**Definition:** stats\_info.h:55

statsinfo::tile\_pos

int tile\_pos

**Definition:** stats\_info.h:46

Info

struct statsinfo Info

stores info needed to create the summary graphs

update\_ACGT\_counts

int update\_ACGT\_counts(uint64\_t \*ACGT\_low, char ACGT)

update, for current tile, ACGT counts.

**Definition:** stats\_info.c:363

update\_QPosTile\_table

void update\_QPosTile\_table(Info \*res, Fq\_read \*seq)

update QPostile table

**Definition:** stats\_info.c:389

statsinfo::ACGT\_pos

uint64\_t \* ACGT\_pos

**Definition:** stats\_info.h:64

statsinfo::QPosTile\_table

uint64\_t \* QPosTile\_table

**Definition:** stats\_info.h:63

statsinfo

stores info needed to create the summary graphs

**Definition:** stats\_info.h:41

statsinfo::read\_len

int read\_len

**Definition:** stats\_info.h:42

read\_info

void read\_info(Info \*res, char \*file)

Read Info from binary file.

**Definition:** stats\_info.c:157

defines.h

Macro definitions.

free\_info

void free\_info(Info \*res)

frees allocated memory in Info

**Definition:** stats\_info.c:142

write\_info

void write\_info(Info \*res, char \*file)

Write info to binary file.

**Definition:** stats\_info.c:202

update\_ACGT\_pos

void update\_ACGT\_pos(uint64\_t \*ACGT\_pos, Fq\_read \*seq)

update ACGT\_pos

**Definition:** stats\_info.c:413

statsinfo::tile\_tags

int \* tile\_tags

**Definition:** stats\_info.h:54

statsinfo::nQ

int nQ

**Definition:** stats\_info.h:44

fq\_read.h

fastq entries manipulations (read/write)

statsinfo::nreads

int nreads

**Definition:** stats\_info.h:47

statsinfo::reads\_MlowQ

uint64\_t \* reads\_MlowQ

**Definition:** stats\_info.h:61

statsinfo::sz\_reads\_MlowQ

int sz\_reads\_MlowQ

**Definition:** stats\_info.h:51

statsinfo::sz\_ACGT\_pos

int sz\_ACGT\_pos

**Definition:** stats\_info.h:53

statsinfo::lowQ\_ACGT\_tile

uint64\_t \* lowQ\_ACGT\_tile

**Definition:** stats\_info.h:57

resize\_info

void resize\_info(Info \*res)

resize Info

**Definition:** stats\_info.c:430

statsinfo::qual\_tags

int \* qual\_tags

**Definition:** stats\_info.h:56

print\_info

void print\_info(Info \*res, char \*infofile)

print Info to a textfile

**Definition:** stats\_info.c:234


---

Generated on Mon Mar 19 2018 23:42:01 for FastqPuri by  

 1.8.14
